# Group A Streptococcus Adsorbed Vaccine: Repeated Intramuscular Dose Toxicity Test in Minipigs

**DOI:** 10.1038/s41598-019-46244-2

**Published:** 2019-07-05

**Authors:** Edilberto Postol, Luiz C. Sá-Rocha, Roney O. Sampaio, Lea M. M. F. Demarchi, Raquel E. Alencar, Maria C. D. Abduch, Jorge Kalil, Luiza Guilherme

**Affiliations:** 1 Heart Institute (InCor), School of Medicine, University of São Paulo, São Paulo, Brazil; 2 Neuroimmunology Laboratory School of Veterinary Medicine and Animal Sciences, University of São Paulo, São Paulo, Brazil; 3 Immunology Investigation Institute, National Institute for Science and Technology, University of São Paulo, São Paulo, Brazil; 4 Clinical Immunology and Allergy Division, School of Medicine, University of São Paulo, São Paulo, Brazil

**Keywords:** Cardiac device therapy, Immunology

## Abstract

*Streptococcus pyogenes* infection continues to be a worldwide public health problem causing various diseases in humans and plays an important role in the pathogenesis of rheumatic fever and rheumatic heart disease. We developed a vaccine candidate to prevent S. pyogenes infections, identified as StreptInCor, that presented promising results in mouse models. A certified and independent laboratory conducted two repeated intramuscular dose toxicity tests (28 days, four weekly injections). The first test, composed of four experimental groups treated with 0 (vehicle), 50, 100 or 200 µg/500 µL StreptInCor, did not show significant alterations in clinical, hematological, biochemical or anatomopathological parameters related to the administration of StreptInCor. In addition to the parameters mentioned above, we evaluated the cardiac function and valves of animals by echocardiography before and after administration of 200 µg/500 µL StreptInCor versus placebo. We did not observe any changes related to StreptInCor administration, including changes in cardiac function and valves in animals, after receiving the highest dose of this vaccine candidate. The results obtained in the two repeated intramuscular dose toxicity tests showed that this vaccine formulation did not induce harmful effects to the tissues and organs studied, indicating that the candidate vaccine is well tolerated in minipigs.

## Introduction

*Streptococcus pyogenes*, also known as group A streptococci (GAS), are major human bacterial pathogens and are responsible for several diseases in humans. These bacteria may cause different clinical manifestations, from a sore throat to necrotizing fasciitis and toxic shock syndrome. Nonsuppurative sequelae may also occur following GAS infections, such as glomerulonephritis and rheumatic fever (RF). RF may occur after improperly treated streptococcal pharyngitis in susceptible individuals and can affect the skin, joints, brain and heart. Among RF sequelae, rheumatic heart disease (RHD) is the most important and affects approximately 30 to 45% of patients with RF. Approximately 616 million new cases of pharyngitis occur each year, with at least 517,000 deaths from more serious diseases due to GAS infections, such as RHD, acute post-streptococcal glomerulonephritis and invasive GAS diseases^1^. Penicillin-benzathine is the treatment of choice to prevent new GAS infections and RF recurrences (primary and secondary prophylaxis)^2^. However, primary and secondary prevention efforts are expensive. The incidence of GAS disease is high, especially in developing countries due to the low levels of economic development and precarious conditions of public health and sanitation. In Brazil RF/RHD accounts for 90% of cardiac surgeries in children.

RF and RHD begins in childhood, usually in children living in poor health and housing conditions, associated with poverty, which facilitates the emergence of recurrent infections by GAS. Although the pathogenesis of RHD is not yet fully understood, there is evidence indicating that the cross reactivity between streptococcal M protein and cardiac proteins is involved in triggering the disease^3,4^.

Several studies show the existence of genes related to RF and RHD susceptibility in the host. In this sense, several polymorphisms in genes encoding immunity-related proteins have been described. Among these, the class II leukocyte antigen (HLA class II) molecules that are present in antigen-presenting cells (APCs) play a central role in the activation of T lymphocytes and consequent activation of the immune response^5–9^.

On the other hand, we have the rheumatogenic strains of GAS. Streptococcal M protein is the major surface protein and the more antigenic antigen. It is composed of two α-helix polypeptide chains anchored in the S. pyogenes cell wall and is extremely polymorphic and antigenic in its N-terminal region, which defines the different serotypes of GAS and the C-terminal region that is highly conserved among the different serotypes of GAS^10^. Typing of GAS strains is based on the nucleotide sequence that defines the hypervariable region of the M protein (*emm*-typing) and is able to distinguish more than 220 different *emm* types. Of these, some are more often associated with the development of RHD^11,12^.

The development of a GAS vaccine may bring numerous benefits, which might prevent not only streptococcal pharyngitis but also invasive infections, as well as the emergence of new cases of RF and its complications. Since pioneering studies by Lancefield^13^, M protein is the target of choice for the production of a GAS vaccine. Some studies have described N- and C-terminal M-protein-based vaccine^14,15^, with promising results when tested in clinical trials^16,17^.

The StreptInCor candidate vaccine contains 55 synthetic amino acid residues of the M protein C-terminal region. Aluminum hydroxide-diluted StreptInCor, tested on inbred, outbred and transgenic mice harboring human HLA class II alleles, has been shown to be an excellent immunogen with no cross-reaction with cardiac proteins and no deleterious effects in vaccinated animals^18,19^. Based on these data, an independent laboratory performed and conducted preclinical evaluation in rodent and nonrodent. Minipigs were chosen as the nonrodent species in our study because of their anatomical, physiological and biochemical similarities with humans, especially those related to the cardiovascular system^20^. Two repeated dose experiments in minipigs were performed to verify possible toxic effects as well as signs of heart injuries in response to the administration of our vaccine candidate in these animals.

## Results

### Experimental design

After two weeks of acclimatization, the minipigs received 4 doses at seven-day intervals of placebo or the formulations containing increasing concentrations of StreptInCor (50, 100 or 200 µg/500 µL) by intramuscular injection. Seven days after the last administration of the formulations we collected blood samples for hematological and biochemical evaluation as well as titration of antibodies produced against StreptInCor. Urine samples were also collected for analysis. The animals were euthanized and then necropsied for the macroscopic evaluation of the organs and subsequent microscopic study of the same. Veterinarians followed the animals throughout the experimental period. The second experiment used only two groups (placebo and 200 µg/500 µL) and had echocardiography exams added three days prior to the administration of the formulations and three days before minipigs euthanasia. The Supplementary Fig. S1 shows the flow chart of the entire experimental design.

### Clinical follow-up

No mortality was recorded during the experimental period. In addition, the veterinarians did not observe clinical and behavioral changes related to vaccine administration throughout the experimental period in the first and second experiments.

### Body weight, food and water consumption

**Figure 1:**
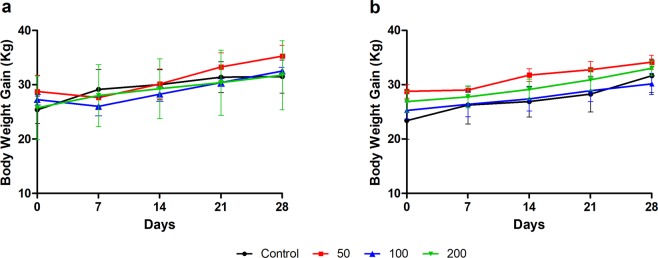
Weight measurements of male (**a**) and female (**b**) minipigs treated with placebo or increasing concentrations of StreptInCor in the formulations of the GAS adsorbed vaccine (50, 100, or 200 µg/500 µL).

The increase in body weight of all animals observed during the experiment was consistent with the food and water intake in the experimental period (see Supplementary Tables S1 and S2). All groups presented a similar weight curve to each other (Fig. 1).

### Hematological, biochemical and urine analyses

Evaluation of blood samples showed that the administration of different amounts of StreptInCor (50, 100 or 200 µg) into vaccine doses (500 µL) did not interfere with the hematological parameters analyzed (Table 1), except for a lower eosinophil value in the female placebo group than in the female group receiving the lowest dose (50 µg/500 µL).

**Table 1:** Hematological data from the male and female minipigs after administration of placebo or StreptInCor at different doses (50, 100, or 200 µg/500 µL).

	Placebo	50 µg	100 µg	200 µg
**Males**				
RBC (10^12^/L)	7.9 ± 0.3	7.8 ± 0.5	7.6 ± 1.0	8.0 ± 0.4
Hemoglobin (g/L)	131.0 ± 3.7	130.3 ± 9.5	123.8 ± 13.4	127,0 ± 7.9
Hematocrit (fraction)	0.4 ± 0.02	0.4 ± 0.02	0.4 ± 0.04	0.4 ± 0.02
MCV (fL)	53.1 ± 2.5	54.5 ± 1.1	54.0 ± 1.5	52.5 ± 2.4
MHC (pg)	16.5 ± 0.4	16.7 ± 1.3	16.4 ± 0.6	15.8 ± 0.4
MCHC (g/L)	311.5 ± 8.0	305.8 ± 18.7	303.4 ± 4.5	300.6 ± 9.6
Platelets (10^9^/L)	404 ± 120	442 ± 113	390 ± 59	378 ± 39
Leukocytes (10^9^/L)	21.4 ± 1.0	17.1 ± 3.2	18.2 ± 1.7	16.5 ± 1.0
Lymphocytes (fraction)	0.4 ± 0.03	0.4 ± 0.01	0.4 ± 0.02	0.5 ± 0.03
Neutrophils (fraction)	0.3 ± 0.004	0.3 ± 0.03	0.3 ± 0.02	0.3 ± 0.06
Monocytes (fraction)	0.1 ± 0.01	0.2 ± 0.04	0.1 ± 0.02	0.1 ± 0.02
Eosinophils (fraction)	0.05 ± 0.02	0.05 ± 0.01	0.06 ± 0.01	0.06 ± 0.01
Basophils (fraction)	0.02 ± 0.01	0.01 ± 0.01	0.01 ± 0.002	0.01 ± 0.01
**Females**				
RBC (10^12^/L)	7.6 ± 0.6	8.1 ± 0.3	6.9 ± 0.4	8.7 ± 0.5
Hemoglobin (g/L)	122.3 ± 7.4	124.8 ± 5.1	109.3 ± 5.2	124.8 ± 15.0
Hematocrit (fraction)	0.4 ± 0.05	0.4 ± 0.01	0.4 ± 0.01	0.4 ± 0.03
MCV (fL)	58.2 ± 9.1	51.6 ± 0.9	53.3 ± 1.4	49.7 ± 5.4
MHC (pg)	16.2 ± 0.7	15.4 ± 0.2	15.9 ± 0.3	14.4 ± 2.3
MCHC (g/L)	283.3 ± 42.0	298.9 ± 5.7	298.2 ± 3.6	288.5 ± 24.8
Platelets (10^9^/L)	398 ± 40	362 ± 51	407 ± 48	378 ± 3
Leukocytes (10^9^/L)	19.2 ± 0.7	16.6 ± 2.1	18.2 ± 5.8	18.4 ± 1.3
Lymphocytes (fraction)	0.5 ± 0.02	0.4 ± 0.05	0.5 ± 0.05	0.5 ± 0.02
Neutrophils (fraction)	0.4 ± 0.03	0.3 ± 0.05	0.3 ± 0.05	0.3 ± 003
Monocytes (fraction)	0.1 ± 0.03	0.2 ± 0.03	0.1 ± 0.04	0.1 ± 0.02
Eosinophils (fraction)	0.03 ± 0.01	0.07 ± 0.02*	0.06 ± 0.01	0.05 ± 0.004
Basophils (fraction)	0.01 ± 0.01	0.01 ± 0.004	0.01 ± 0.003	0.01 ± 0.003

Each value represents the mean ± SD between four animals per group. RBC, red blood cells; MCV, mean cell volume; MCH, mean cell hemoglobin; MCHC, mean cell hemoglobin concentration. Dunn’s Multiple Comparison test: *significant difference between in eosinophil values compared to the placebo group, *p* < 0.05.

Clinical chemistry data, presented in Table 2, did not show significant differences among the experimental groups. The urine sample analysis also showed similarity among all the experimental groups, without significant alterations in the different urinalysis items evaluated (see Supplementary Tables S3–S6).

**Table 2:** Serum biochemistry data from the male and female minipigs after the administration of placebo or StreptInCor at different doses (50, 100, or 200 µg/500 µL).

	Placebo	50 µg	100 µg	200 µg
**Males**				
Urea (mmol/L)	7.5 ± 1.6	8.1 ± 1.7	9.1 ± 0.7	8.3 ± 0.9
Creatinine (µmol/L)	134.9 ± 13.6	155.2 ± 7.5	144.6 ± 27.6	152.4 ± 21.5
AST (IU/L))	75.3 ± 23.4	92.8 ± 39.3	84.5 ± 16.4	65.3 ± 19.4
ALT (IU/L)	53.0 ± 8.0	59.8 ± 9.1	60.5 ± 15.4	76.7 ± 4.8
Glucose (mmol/L)	5.3 ± 0.6	4.2 ± 0.4	4.6 ± 0.9	5.0 ± 0.8
Cholesterol total mmol/L)	1.8 ± 0.15	1.8 ± 0.27	1.6 ± 0.34	1.5 ± 0.08
Albumin (g/L)	27.7 ± 6.5	27.6 ± 2.2	27.7 ± 5.7	27.3 ± 2.7
Globulin (g/L)	31.1 ± 3.1	38.6 ± 4.6	38.5 ± 6.9	33.5 ± 2.4
Total Protein (g/L)	58.7 ± 6.1	66.2 ± 6.7	66.2 ± 4.6	60.8 ± 4.1
Phosphorus (mmol/L)	7.9 ± 1.4	7.7 ± 1.7	8.0 ± 0.8	7.1 ± 0.5
Chloride (mmol/L)	97.2 ± 2.0	103.0 ± 1.8	94.3 ± 5.2	96.4 ± 4.7
Calcium (mmol/L)	2.7 ± 0.1	2.7 ± 0.3	2.7 ± 0.1	2.8 ± 0.2
**Females**				
Urea (mmol/L)	10.4 ± 1.3	10.6 ± 2.7	14.1 ± 1.4	12.0 ± 1.7
Creatinine (µmol/L)	152.5 ± 9.2	181.7 ± 11.6	164.1 ± 16.7	170.7 ± 14.6
AST (IU/L))	85.3 ± 6.3	75.5 ± 17.4	62.5 ± 35.6	97.8 ± 15.7
ALT (IU/L)	72.7 ± 9.2	62.2 ± 20.5	72.8 ± 39.9	72.8 ± 11.3
Glucose (mmol/L)	4.2 ± 0.9	4.7 ± 1.6	4.5 ± 0.9	6.2 ± 1.5
Cholesterol total (mmol/L)	1.5 ± 0.04	1.8 ± 0.19	1.7 ± 0.26	1.8 ± 0.23
Albumin (g/L)	30.5 ± 1.7	29.8 ± 5.7	30.3 ± 2.7	30.3 ± 4.5
Globulin (g/L)	28.2 ± 5.2	36.8 ± 3.0	36.8 ± 1.2	39.8 ± 16.6
Total Protein (g/L)	58.6 ± 3.9	66.5 ± 8.2	67.2 ± 1.6	70.1 ± 17.1
Phosphorus (mmol/L)	7.7 ± 0.7	7.7 ± 0.6	7.9 ± 0.6	7.4 ± 0.5
Chloride (mmol/L)	100.0 ± 3.6	101.2 ± 3.7	96.6 ± 1.4	97.4 ± 5.8
Calcium (mmol/L)	2.6 ± 0.2	2.7 ± 0.2	2.7 ± 0.2	2.8 ± 0.2

Each value represents the mean ± SD between four animals per group. AST- aspartate aminotransferase; ALT-alanine aminotransferase.

### Serum antibody measurement

The evaluation of humoral responses against StreptInCor after the four administrations of the formulations used in this study did not show significant differences between animals receiving placebo and those receiving increasing concentrations of StreptInCor (see Supplementary Fig. S2). However, it was possible to see an increase in anti-StreptInCor antibodies in two females in the group receiving the lowest dose (50 µg/500 µL).

### Necropsy, relative organs weight determination and histopathological analysis

**Figure 2:**
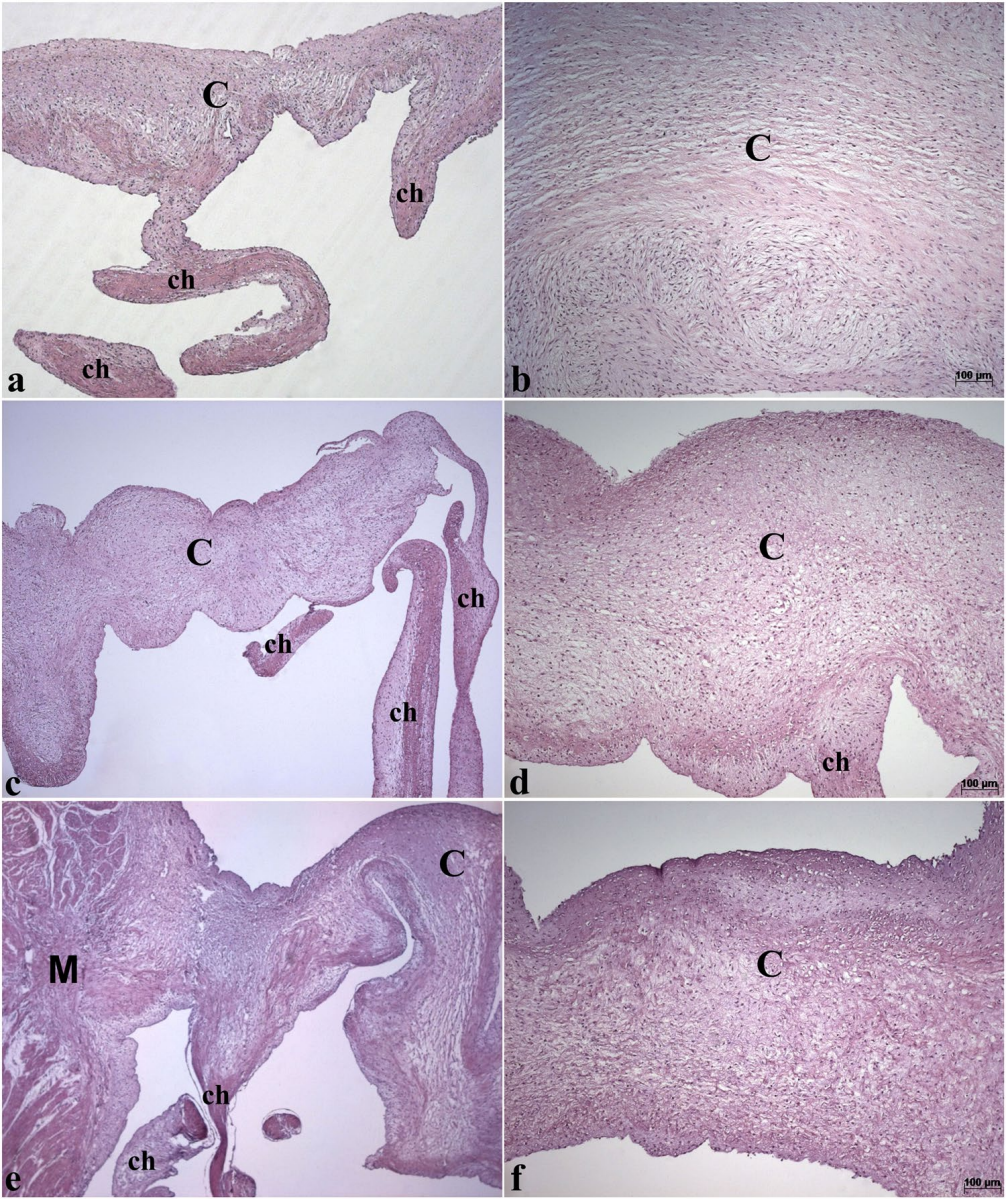
Photomicrographs of histological sections of mitral valve from male minipigs: (**a,b**) control group treated with placebo; (**c,d**) group treated with StreptInCor intermediate dose (100 µg/500 µL); (**e,f**) group treated with higher dose (200 µg/500 µL) of StreptInCor. Hematoxylin and eosin staining are showed (**a,c,e**) (x50) and (**c,d,f**) (x100). v = valve cusp; ch = tendinous cords and M = myocardium.

Table 3 shows the weights of the organs relative to the animal body weight that was registered prior to euthanasia. We observed a significant difference (p < 0.05) between the relative adrenal weights among the females of the placebo group (increased) in relation to those of the lowest and highest concentration experimental groups (50 µg and 200 µg/500 µL). We also found a significant difference (p < 0.05) between the relative weights of the ovaries of the placebo group (decreased) and those of the females receiving the intermediate dose of the vaccine (100 µg/500 µL). Despite these observations, histologic evaluation of these organs did not show any abnormalities. One of the veterinarians considered that there was a slight thickening in the free edge of mitral valve at macroscopic examination of one male from experimental group receiving 100 µg/500 µL (Supplementary Fig. S3) when compared to a control one (Supplementary Fig. S4). Nevertheless, valvular and myocardium microscopic analysis of this animal did not show any pathologic alterations (Fig. 2). The right kidney of a female from placebo group weighed 68 g and showed some cortical cysts. The left kidney of the same animal did not show cysts and weighed 38 g. Finally, the liver of a male from placebo group had a mild hepatic congestion (Supplementary Fig. 5). Necrosis, inflammatory infiltrate, fibrosis, neovascularization or other remarkable gross or histopathologic alterations were not found in any organ of all animals from experimental groups.

**Table 3:** Relative organ weight (weight organ as proportion to the total body weight of each animal) from the male and female minipigs after the administration of placebo or StreptInCor at different doses (50, 100, or 200 µg/500 µL).

	Placebo	50 µg	100 µg	200 µg
**Males**				
Heart	0.39 ± 0.02	0.37 ± 0.04	0.39 ± 0.01	0.50 ± 0.09
Liver	2.05 ± 0.11	1.85 ± 0.13	2.01 ± 0.16	2.04 ± 0.51
Kidney	0.17 ± 0.02	0.14 ± 0.01	0.15 ± 0.01	0.15 ± 0.04
Adrenal (10^−3^)^a^	4.46 ± 0.93	4.17 ± 0.58	4.61 ± 1.22	3.16 ± 0.61
Spleen	0.15 ± 0.03	0.15 ± 0.03	0.14 ± 0.02	0.14 ± 0.03
Epydidimis^a^	0.064 ± 0.011	0.064 ± 0.018	0.058 ± 0.026	0.049 ± 0.009
Testicles^a^	0.17 ± 0.03	0.19 ± 0.07	0.18 ± 0.11	0.15 ± 0.03
**Females**				
Heart	0.40 ± 0.03	0.39 ± 0.04	0.38 ± 0.04	0.42 ± 0.08
Liver	2.07 ± 0.11	1.65 ± 0.11	2.17 ± 0.17	1.96 ± 0.20
Kidney	0.16 ± 0.03	0.15 ± 0.01	0.16 ± 0.02	0.14 ± 0.01
Adrenal (10^−3^)^a^	5.30 ± 1.64	3.63 ± 0.61*	3.89 ± 0.47	3.56 ± 1.23*
Spleen	0.16 ± 0.01	0.16 ± 0.01	0.17 ± 0.04	0.13 ± 0.01
Uterus	0.12 ± 0.04	0.09 ± 0.02	0.22 ± 0.09	0.10 ± 0.02
Ovary^a^	4.39 ± 1.16	6.06 ± 0.97	7.50 ± 2.51**	4.56 ± 1.67

Each value represents the mean ± s.d. between four animals per group. *Significant difference (*p* < 0.05) between adrenal relative weights from female groups receiving 50 µg and 200 µg doses of StreptInCor when compared to female control group. **Very significant difference (0.001 < *p* < 0.01) between ovary relative weights from 100 µg experimental female group when compared to female control group. ^a^Data that passed normality test and submitted to one way ANOVA test and Tukey´s Multiple Comparison post test.

### Second experiment and echocardiography exams

As observed in the first experiment, the data obtained from the groups of the second experiment (placebo and 200 µg/500 µL) were similar to each other. In this sense, the control and highest dose groups showed similar weight increases, consistent with the recorded food and water consumption, and the clinical follow-up did not identify any changes in the animals during the experiment. The results of hematological and clinical chemistry tests showed no change suggestive of any damage caused by repeated administration of the vaccine in high doses.

We observed similar relative weights of the organs between the animals of the two groups. The only exception was the difference (p < 0.05) between the relative weights of the kidneys of the females of the 200 µg/500 µL group (0.13% ± 0.01) in relation to those of the placebo group (0.14% ± 0.01). However, no signs of damage (macro- or microscopic) were seen in any organs of the minipigs in either group.

**Figure 3:**
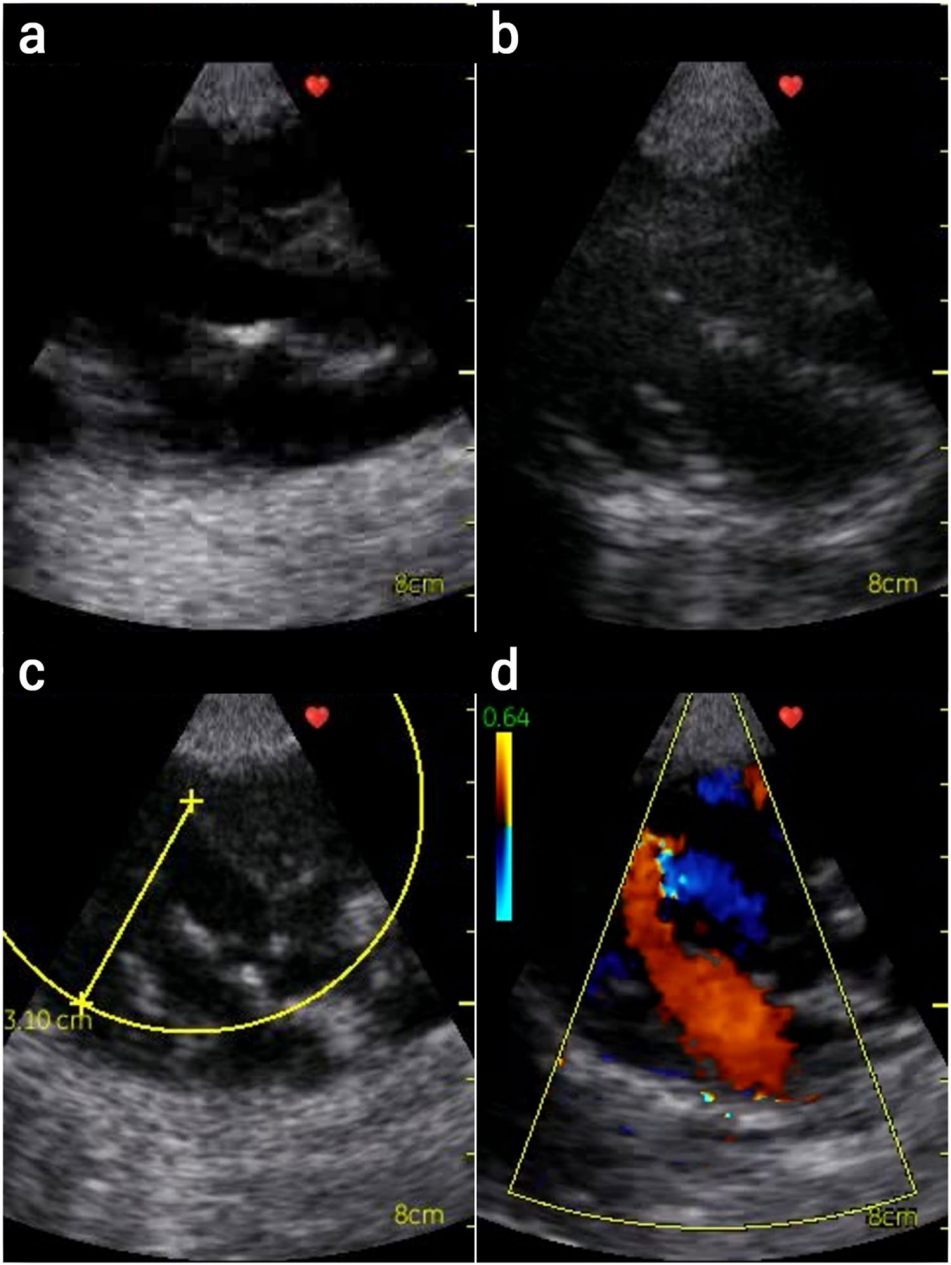
Echocardiography from minipigs before injection of GAS adsorbed vaccine: (**a**) closed mitral valve; (**b**) open mitral valve; (**c**) open mitral valve and measurement of the left ventricle (LV) and closed aortic valve; (**d**) LV color Doppler inflow (red) and outflow (blue).

**Figure 4:**
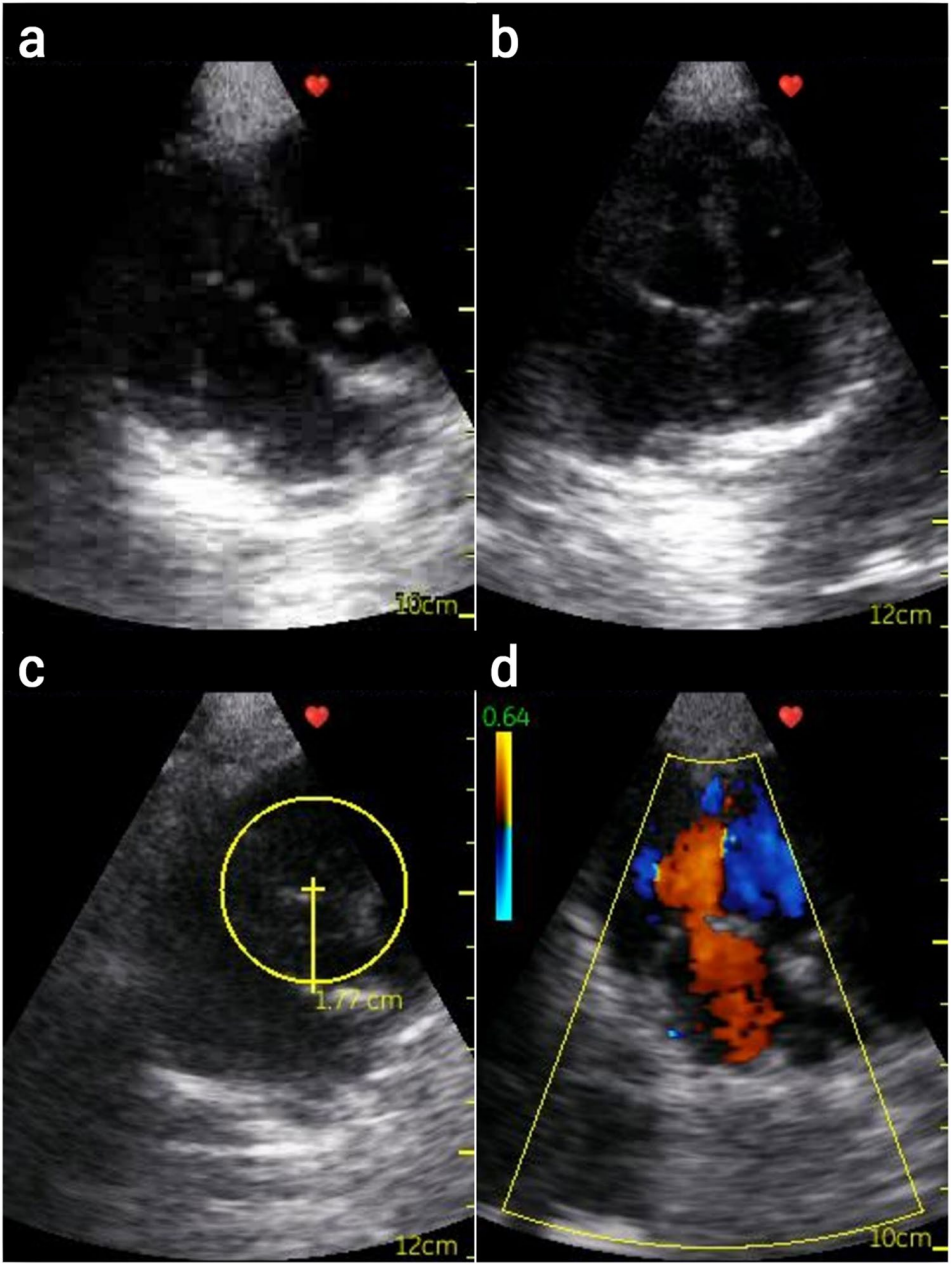
Echocardiography from minipigs after injection of GAS adsorbed vaccine (200 µg/500 µL): (**a**) partially open mitral valve and closed aortic valve; (**b**) four chamber view of mitral and tricuspid valves; (**c)** measurement of the left ventricle (LV) outflow tract; (**d**) LV color Doppler inflow (red) and outflow (blue).

The echocardiography exams showed that the treatment with 200 µg/500 µL StreptInCor did not induce heart alterations compared to the heart condition of the animals before the treatment (Table 4). This result was similar to that observed among minipigs receiving placebo. Figures 3 and 4 show the echocardiography of a minipig before and after the treatment with 200 µg/500 µL StreptInCor, respectively. The supplementary short videos show echocardiography images from short axis view, four chamber view and valves inflow and/or outflow from animals before and after vaccination. No significant changes were observed before (Supplementary Video S1) and after the last administration of the vaccine candidate (Supplementary Video S2).

**Table 4:** Echocardiography data of male and female minipigs before and after administration of placebo or StreptInCor (200 µg/500 µL).

	Echocardiography Observation	Placebo		200 µg	
		Before Treatment	After Treatment	Before Treatment	After Treatment
**Males**					
Systolic Function	^a,b^normal	100%	100%	100%	100%
Mitral Valve	^a,b^normal	75%	100%	100%	100%
	^a^minimal thickness/^b^thickness	25%	—	—	—
Aortic Valve	^a,b^normal	75%	100%	100%	100%
	^a,b^thickness	25%	—	—	—
Echo Acoustic Window	^a^good/^b^adequate	50%	50%	50%	75%
	^a,b^adequate	25%	25%	50%	25%
	^a^adequate/^b^poor	25%	25%	—	—
**Females**					
Systolic Function	^a,b^normal	100%	100%	100%	100%
Mitral Valve	^a,b^normal	100%	100%	100%	100%
Aortic Valve	^a,b^normal	100%	100%	100%	100%
Echo Acoustic Window	^a^good/^b^adequate	100%	75%	75%	50%
	^a,b^adequate	—	—	—	50%
	^a^adequate/^b^poor	—	25%	25%	—

Observations made by ^a^cardiologist and ^b^veterinarian.

## Discussion

This study is part of our project to develop an effective and safe vaccine against *S pyogenes*, capable of inhibiting bacterial colonization and infections in the oropharynx. We also expect a protective effect against more severe manifestations such as toxic shock syndrome and RF/RHD. After promising results obtained in animals^18,19,21^, for the continuity of the project it is necessary to present data obtained in non-clinical safety tests for our national regulatory agency (ANVISA). These studies should be presented prior to Phase I clinical trial. The data presented here refer to the study of toxicity of repeated doses (28 days) performed in minipigs and are important for obtaining data that show possible toxic effects of the vaccine on the physiology, hematology and biochemistry of the animals. It is also possible to detect anatomical and histopathological changes in treated animals and the identification of target organs. Because of the different factors that influence toxicological studies, which go beyond anatomy, it is also necessary to use animals of both sexes in equal numbers^22,23^.

We chose to use minipigs as a nonrodent species for toxicology tests with the StreptInCor candidate vaccine. Minipigs have been increasingly used in toxicological studies for safety assessment as an alternative to dogs and primates, especially in Canada, the European Community and the United States. This is due to physiological, anatomical and genetic similarities to humans, which may contribute to the development of new products for human use. In addition, the anatomy and physiology of cardiovascular system is very similar in humans and minipigs making the later interesting for the evaluation of possible deleterious effects on the heart^20,24–26^. However, background lesions in toxicological tests carried out on minipigs of the Göttingen, Hanford and Yucatan strains in North America and the European Union were described. In a study that evaluated tests performed between 2004 and 2015, the presence of spontaneous inflammatory cellular infiltrates in the heart, liver and kidneys was observed among other findings^27^. Although we do not have information about background changes in the minipigs used in these toxicological tests, this possibility should be considered.

The hematological and sera biochemistry data observed in this study show that there were no significant differences between the groups of minipigs treated with the vehicle and those treated with StreptInCor, considering the variation of the reference values between the different lineages, origins and ages of swine observed in other studies^28–35^. The concentration of eosinophils however was significantly lower in the group of females receiving only aluminum hydroxide compared with females receiving StreptInCor (50 µg/500 µL). Although the mechanism of action of aluminum hydroxide is not fully understood, this adjuvant induces neutrophils and monocytes as well as eosinophils to the site of inoculation following intramuscular injection^36,37^. Eosinophils in turn are able to secrete preformed IL-4 and may play an immunomodulatory role in the activation of B cells elicited by aluminum hydroxide^38,39^. Therefore, we can expect the increase in circulating eosinophils observed in all groups evaluated, with the exception of the female group receiving the vehicle, due to the inoculations of aluminum hydroxide.

The most common background change observed in kidneys of Göttingen, Hanford and Yucatan minipigs is the presence of inflammatory cell infiltrates. Mineralization was also observed in the kidneys of Göttingen minipigs27. The renal cysts observed at necropsy in one female from the control group could be considered as a background lesion. Mild hepatic congestion observed in a male from the placebo was considered a non-specific alteration by the veterinarians, probably a result of the terminal anesthesia process. Histopathological evaluation of kidneys and liver from these animals did not show any alteration suggesting a toxic effect that could be related to the substance applied (placebo)

Since RHD is one of the most severe complications of GAS infections^40,41^, the heart is a possible target organ for adverse reactions during the administration of a vaccine based on M protein. Considering this, an accurate anatomopathologic examination of the heart, with special attention to cardiac valves was performed. No remarkable gross or histologic alterations were found in cardiac valves, myocardium or other cardiac tissues from animals of all experimental groups.

Cross-reactive antibodies play a key role in the pathogenesis of RF and RHD^40,42^. Thus, it is important to demonstrate that antibodies produced against SI do not cross-react with human antigens. We have previously demonstrated that isogenic, outbred and transgenic mice (human HLA class II carriers) did not cross-react and did not present deleterious effects after immunizations^18,19^. We did not observe a good production of anti-StreptInCor antibodies except in two females from experimental group receiving 50 µg/500 µL (Supplementary Fig. S2). In this intramuscular repeated dose toxicity study, the vaccine administrations were performed at one-week intervals. This protocol is performed for the characterization of the toxicological profile of the substance by repeated administration. However, continuous exposure of the animals to an immunogen does not result in increased circulating antibodies^43,44^.

To confirm the absence of background changes in the heart that may increase in severity during a toxicity test as well as possible adverse effects caused by repeated administration of the vaccine, we decided to do a second experiment evaluating animals treated with placebo with the highest doses of StreptInCor (200 µg/500 µL). In this experiment, we performed echocardiography before and after the administration of the vaccine. The use of echocardiography was recently used in a rat model for RHD^45^. The authors were able to observe valvar fibrosis with nodules in rats treated with whole-killed group G streptococci similar to GAS M5 (a rheumatogenic strain). In contrast, the control rats presented uniform valvar structures. These results were later confirmed with histological findings, showing the usefulness of this imaging technique for *in vivo* evaluation of possible alterations before and after treatment of the animals. In our experiment, no echocardiographic alterations were observed in any minipig treated with placebo or 200 µg/µL StreptInCor groups in our second experiment. No lesions or deleterious reactions to StreptInCor were found in these animals after macroscopic and histopathological examination at necropsy.

In summary, the results of our toxicity study indicated that the vaccine formulation containing StreptInCor did not induce harmful effects on different tissues and organs of minipigs and was therefore a safe and well-tolerated vaccine candidate in these animals.

However, as widely scientifically known, GAS is a human exclusive pathogen. Although our animal studies indicate that StreptInCor is a safe vaccine candidate, there is not possible to be completely sure about its safety before a phase I/II clinical study is initiated, which is planned for a near future.

A phase I/IIa clinical study will be the next step and will aim to assess the safety and immunogenicity of our vaccine candidate. A randomized, double-blind, placebo-controlled, dose escalation phase I/IIa clinical trial will aim to assess the safety and immunogenicity of StreptInCor in volunteers. To do this, we intend to have four groups consisting of twelve healthy adults, each group receiving the vaccine in three different concentrations of StreptInCor (50, 100 or 200 µg/500 µL) formulated with aluminum hydroxide as a vaccine adjuvant or placebo. The volunteers will randomly receive two doses of vaccine at 28-day intervals plus a booster 6 months after the initial vaccination.

## Methods

TECAM Tecnologia Ambiental (São Roque, SP, Brazil) conduced the repeated intramuscular dose toxicity tests (28 days) under conditions of good laboratory practice (GLP) and following national^46^ and international standards^47,48^. All procedures were in accordance with national guidelines of the National Animal Experiment Control Council (CONCEA) and Committee for Animal Care and Use (COBEA) and international requirements based on the “Guide for the Care and Use of Laboratory Animals”^49^. The animals were kept in a facility accredited by Association for Assessment and Accreditation of Laboratory Animal Care (AAALAC, CIAEP/CONCEA 01.0242.214), and approved by Tecam Ethical Committee for Animal Research (study 5518/2015IM, protocol 31).

### Test substance

The synthetic peptide StreptInCor was produced by Polypeptide Laboratories (Torrance, CA, USA) using good manufacturing practice (GMP) standards. The Butantan Institute (São Paulo, Brazil) prepared the vaccine solution, diluting StreptInCor into aluminum hydroxide as follows: 50 µg/500 µL, lot 15055 (low dose); 100 µg/500 µL, lot 15056 (medium dose); and 200 µg/500 µL, lot 15057 (high dose). A placebo formulation (aluminum hydroxide and saline) was lot 15054.

### Animals

Male and nulliparous non-pregnant female minipigs (*Sus scrofa domesticus*), belonging to the BR-1 colony, that were 6- to 8-month-old at the beginning of the study were purchased from Minipig Pesquisa e Desenvolvimento (Campina do Monte Alegre, SP, Brazil). Each minipig was evaluated and weighed by a veterinarian, and only healthy animals were selected for the study. They were identified by ear tags and housed into pigpens where they were acclimated for fourteen days before the experiments.

The environment was monitored during the entire test period, and the temperature averages were 24.5 °C. In addition, they stayed under a 12-hr light/dark cycle, receiving species-specific rations (Purina, Ribeiro Preto, SP, Brazil) and filtered water *ad libidum*.

### Administration of vaccine doses

Eight minipigs, four males and four nulliparous and non-pregnant females, per group were treated by intramuscular injection at doses of 50, 100 or 200 µg/500 µL StreptInCor or with placebo (vehicle) at days 0, 7, 14 and 21 (first experiment). The animals had their posterior limb trichotomized, where they received the injections (posterior muscle, 500 µL) with the respective formulations. To evaluate the minipig hearts *in vivo* by echocardiography, two additional groups, placebo and 200 µg/µL StreptInCor, were used in a second experiment following the same protocol described for the first experiment. The Supplementary Fig. S1 shows the study design used in these experiments.

### Clinical follow-up

The animals were monitored individually throughout the acclimation as well as during the experimental period. Experienced veterinarians classified the severity of clinical signs as mild, moderate or severe based on physical examination of the animals. Clinical observations included changes in the fur, skin, eyes, mucous membranes, occurrence of secretions and excretions and autonomic activity (lacrimation, piloerection, changes in the pupils, respiratory pattern). The veterinarians were also aware of possible changes in gait, posture and reaction to manipulation, as well as the presence of tonic or clonic movements and stereotypies such as excessive grooming, repetitive circulatory movements, self-mutilation and walking backwards. Finally, local tolerance was assessed by the presence of edema, erythema, desquamation, wound, alopecia and any other signs of local irritation and/or inflammation.

The body weight of the minipigs was measured immediately prior to test product applications and weekly until completion of the study. Food and water intake were also measured, which were calculated in g/animal/day and L/animal/day, respectively.

### Euthanasia

After twenty-eight days, all animals were sedated with an association of xylazine (8 mg/kg) and ketamine (65 mg/kg) injected intramuscularly. Blood samples with (EDTA, for hematological tests) or without anticoagulant (for biochemical tests) were collected through cardiac puncture after deep anesthesia and prior to euthanasia. The veterinarians euthanized the minipigs by intracardiac injection of potassium chloride after confirmation of deep anesthesia.

### Hematological and biochemical analyses

Hematological tests, including red blood cell count, hemoglobin concentration, hematocrit, mean corpuscular volume (MCV), mean corpuscular hemoglobin (MCH), mean corpuscular hemoglobin concentration (MCHC), white blood cell count and platelet count, were evaluated using HEMATOClin 2.8 Vet equipment (QUIBASA Bioclin, Brazil).

Biochemical tests performed using commercial kits included determination of serum levels of glucose, total cholesterol, urea, creatinine, alanine aminotransferase, aspartate aminotransferase, albumin, globulin, total protein, sodium, chloride and potassium. The results were obtained by spectrometry in liquid medium by using semiautomatic TP Analyzer equipment (Thermo-Plate, Brazil).

### Sera antibody measurement

Sera antibody titers were quantified using ELISA. Briefly, we diluted 1 µg of StreptInCor in coating buffer (0.05 M carbonate-bicarbonate, pH 9.6, 50 µl/well) and plated onto a 96-well MaxiSorp assay plate (Nunc, Denmark). After overnight incubation, the plates were blocked with 0.25% (w/v) gelatin (Sigma) and 0.05% (v/v) Tween-20 (Sigma) in PBS (dilution buffer) for 1 h at room temperature. Serial 2-fold dilutions of the sera in dilution buffer, starting at 1:100, were added to the plates (50 µl/well). After a 2-h incubation at 37 °C and three washes (200 µl/well) with 0.05% Tween-20 in PBS (rinse buffer), the plates were incubated with peroxidase-conjugated anti-pig IgG (Sigma, USA) diluted 1:5000 in dilution buffer (50 µL/well) for an hour at 37 °C. Next, the plates were washed three times (200 µl/well) with rinse buffer, and the reaction was carried out with 50 µL/well of 0.4 mg/ml o-phenylenediamine (OPD, Sigma) in 100 mM sodium citrate (Merck, Germany) containing 0.03% H_2_O_2_ (Merck). After 10 minutes at room temperature, the reactions were stopped with 4 N H_2_SO_4_. Endpoint titers were defined as the reciprocal of the highest dilution giving an absorbance higher 0.100 after optical density evaluation using a 490 nm ELISA filter in a Multiskan GO equipment (Thermo Fisher Scientific Oy, Finland). Samples with values less than 0.100 at 1:100 dilution were considered negative.

### Urine analysis

Urine samples were collected by cystocentesis during necropsy. These samples were analyzed by using veterinary colorimetric strips for urinalysis with a VetLab UA Analyzer (IDEXX Laboratories Inc., USA) to obtain the specific gravity, pH, appearance, protein, glucose, bilirubin, ketones and urobilinogen.

### Echocardiography

A cardiologist experienced in echocardiography methods obtained the echocardiographs from minipigs using a VSCAN with Dual Probe ultrasound system (GE VINGMED ULTRASOUND AS, Norway). We obtained the data before the first and after the last administration of test products in animals of the second experiment (placebo and 200 µg/500 µL doses). Acoustic window, mitral and aortic valve anatomy, focusing on leaflet mobility, and the presence of abnormalities such as valve thickening, calcification or congenital diseases were evaluated. Likewise, we evaluated the transvalvular mitral, tricuspid and aortic flows with color Doppler. The cardiologist and a veterinarian experienced in echocardiography independently evaluated the data in a blinded manner. Another independent echocardiographic analysis was requested if a significant difference in their evaluation occurred.

### Necropsy, relative organ weight determination and histopathological analysis

Veterinarians performed necropsies of all animals and collected tissue samples for histological analysis performed necropsies of all animals. The vital organs such as brain, liver, kidneys, adrenal glands, heart, gonads (testis and epididymis or ovary), thymus and spleen were collected and the relative organ weight (weight of organ as percentage to the total body weight of each minipig) was calculated and compared with the relative organ weight of the control group minipigs. After macroscopic evaluation, samples of brain, lung, stomach, esophagus, small intestine, large intestine, liver, pancreas, kidney, adrenal glands, heart, gonad (testis or ovary), urinary bladder, femur, thymus, spleen and lymph nodes were fixed in 10% buffered formalin. These samples were sent to a specialized laboratory for histopathological processing in which they were dehydrated in alcohol baths with a concentration gradient, cleared in xylene and embedded in paraffin blocks. Five-micron sections were cut, mounted on slides and stained by the hematoxylin and eosin technique.

### Statistical analysis

For each analyzed parameter, the data are expressed as the mean ± standard deviation (s.d.) between different animals within each group. All data were examined by the D’Agostino and Pearson omnibus normality test to verify whether the data could be analyzed by parametric tests. When data passed the normality test, we employed one-way analysis of variance and the Tukey post-test to compare all pairs of columns. If the data did not pass the normality test, we used the Kruskal-Wallis test and the Dunn’s Multiple Comparison post-test to compare all pairs of columns. We used GraphPad Prism software version 5.01 for Windows (GraphPad Software, San Diego California USA, www.graphpad.com) to analyze the data and considered *p* values < 0.05 significant.

## Supplementary information

Supplementary Dataset 1
