# Supplementary material for: Group A Streptococcus Adsorbed Vaccine: Repeated Intramuscular Dose Toxicity Test in Minipigs
Source: Sci Rep. 2019 Jul 5;9:9733. doi: 10.1038/s41598-019-46244-2 (PMC6611820; doi:10.1038/s41598-019-46244-2)
Supplement: Supplementary file 1 — Supplementary Dataset 1 [file 41598_2019_46244_MOESM1_ESM.docx]

Group A Streptococcus Adsorbed Vaccine: Repeated Intramuscular Dose Toxicity Test in Minipigs

Edilberto Postol^1,3^, Luiz C. Sá-Rocha^2,3^, Roney O. Sampaio^1,^, Lea M. M. F. Demarchi^1^, Raquel E. Alencar^1,3^, Maria C. D. Abduch^1^, Jorge Kalil^1,3,4^ & Luiza Guilherme^1,3^

^1^Heart Institute (InCor), School of Medicine, University of São Paulo, São Paulo, Brazil. ^2^Neuroimmunology Laboratory School of Veterinary Medicine and Animal Sciences, University of São Paulo, São Paulo, Brazil. ^3^Immunology Investigation Institute, National Institute for Science and Technology, University of São Paulo, São Paulo, Brazil. ^4^Clinical Immunology and Allergy Division, School of Medicine, University of São Paulo, São Paulo, Brazil. Corresponding and requests for materials should be addressed to L.G. (email: luizagui@usp.br)

Supplementary Figure S1: Experimental design of repeated intramuscular dose toxicity test in minipigs.

EXPERIMENTAL GROUPS

04 Male and 04 Female Minipigs/Group

Second Experiment

Placebo - StreptIncor (200 µg/500 µL)

First Experiment

Placebo - StreptInCor (50 - 100 - 200 µg/500 µL)

Acclimatization

from d. -14 to d. 0

Clinical Follow Up

from d. -14 to d. 28

Echocardiograph

d. -3

Administration of Vaccine Doses

d. 0 – d. 7 – d. 14 – d. 21

Blood Sample Collection

d. 28

Euthanasia

d. 28

Hematological, Biochemical Analysis and

Serum Antibody Measurements

Macroscopic Evaluation

Weighting of Organs

d. 28

Body Weight

Food and Water Intake

d. 0 – d. 7 – d. 14 – d. 21 – d.28

Urine Analysis

Echocardiograph

d. 25

Histopathological Analysis

Urine Sample Collection

d. 28

Supplementary Figure S1: Experimental design of repeated intramuscular dose toxicity test in minipigs.

Supplementary Table S1: Weekly food consumption (Kg).

|  | Placebo | 50 µg | 100 µg | 200 µg |
| --- | --- | --- | --- | --- |
| **Males** |  |  |  |  |
| Before 1^st^ injection  (from day -7 to 0) | 7.9 ± 0.2 | 7.7 ± 0.2 | 7.7 ± 0.2 | 8.0 ± 0.2 |
| Before 2^nd^ injection  (from day 0 to 7) | 7.5 ± 0.3 | 7.5 ± 0.2 | 7.4 ± 0.2 | 7.5 ± 0.3 |
| Before 3^rd^ injection  (from day 7 to 14) | 7.8 ± 0.1 | 7.9 ± 0.1 | 7.7 ± 0.2 | 7.8 ± 0.2 |
| Before 4^th^ injection  (from day 14^th^ to 21^st^) | 8.1 ±0.1 | 8.0 ± 0.1 | 8.1 ± 0.0 | 8.2 ± 0.1 |
| Before necropsy  (from day 21^st^ to 28^th^) | 8.4 ± 0.1 | 8.3 ± 0.1 | 8.4 ± 0.2 | 8.5 ± 0.1 |
|  |  |  |  |  |
| **Females** |  |  |  |  |
| Before 1^st^ injection  (from day -7 to 0) | 7.0 ± 0.2 | 6.8 ± 0.1 | 6.6 ± 0.1 | 6.8 ± 0.2 |
| Before 2^nd^ injection  (from day 0 to 7) | 6.5 ± 0.2 | 6.6 ± 0.2 | 6.6 ± 0.3 | 6.6 ± 0.1 |
| Before 3^rd^ injection  (from day 7 to 14) | 7.0 ± 0.3 | 7.0 ± 0.1 | 7.1 ± 0.3 | 7.0 ± 0.2 |
| Before 4^th^ injection  (from day 14^th^ to 21^st^) | 7.3 ±0.2 | 7.3 ± 0.2 | 7.2 ± 0.2 | 7.3 ± 0.2 |
| Before necropsy  (from day 21^st^ to 28^th^) | 7.3 ± 0.2 | 7.4 ± 0.2 | 7.2 ± 0.1 | 7.5 ± 0.1 |

Each value represents the mean ± SD between four animals per group.

Supplementary Table S2: Weekly water consumption (L).

|  | Placebo | 50 µg | 100 µg | 200 µg |
| --- | --- | --- | --- | --- |
| **Males** |  |  |  |  |
| Before 1^st^ injection  (from day -7 to 0) | 10.3 ± 0.4 | 10.5 ± 0.5 | 9.7 ± 1.2 | 10.0 ± 1.2 |
| Before 2^nd^ injection  (from day 0 to 7) | 10.9 ± 0.5 | 10.5 ± 0.6 | 10.2 ± 0.9 | 10.1 ± 1.3 |
| Before 3^rd^ injection  (from day 7 to 14) | 10.8 ± 0.3 | 10.4 ± 1.3 | 10.0 ± 1.2 | 9.9 ± 1.1 |
| Before 4^th^ injection  (from day 14^th^ to 21^st^) | 11.1 ±0.4 | 11.0 ± 0.6 | 10.2 ± 0.8 | 10.5 ± 1.1 |
| Before necropsy  (from day 21^st^ to 28^th^) | 11.0 ± 0.2 | 11.2 ± 0.5 | 10.2 ± 1.0 | 10.7 ± 0.6 |
|  |  |  |  |  |
| **Females** |  |  |  |  |
| Before 1^st^ injection  (from day -7 to 0) | 8.7 ± 0.4 | 8.7 ± 0.4 | 8.8 ± 0.5 | 8.9 ± 0.3 |
| Before 2^nd^ injection  (from day 0 to 7) | 8.6 ± 0.5 | 9.0 ± 0.6 | 8.7 ± 0.6 | 8.8 ± 0.6 |
| Before 3^rd^ injection  (from day 7 to 14) | 9.2 ± 0.5 | 8.9 ± 0.9 | 8.6 ± 0.5 | 8.3 ± 0.5 |
| Before 4^th^ injection  (from day 14^th^ to 21^st^) | 9.1 ±0.7 | 8.9 ± 0.4 | 8.7 ± 0.6 | 8.6 ± 0.5 |
| Before necropsy  (from day 21^st^ to 28^th^) | 9.6 ± 0.8 | 9.1 ± 0.5 | 8.5 ± 0.6 | 9.1 ± 0.7 |

Each value represents the mean ± SD between four animals per group.

Supplementary Table S3. Urine analysis of male minipigs treated with placebo or StreptInCor at the concentration of 50 µg/500 µL

| Animal (male) | #1 | #2 | #3 | #4 |
| --- | --- | --- | --- | --- |
| **Placebo:** |  |  |  |  |
| Specific gravity | 1.020 | 1.015 | 1.015 | 1.020 |
| pH | 8.0 | 7.0 | 5.0 | 7.5 |
| Appearance | clean/transparent | slightly cloudy | clean/transparent | clean/transparent |
| Protein | - | + | - | - |
| Glucose | - | ++ | - | - |
| Bilirubin | - | - | - | - |
| Ketones | - | - | - | - |
| Leukocytes | - | - | - | - |
| Urobilinogen | +/- | - | - | +/- |
|  |  |  |  |  |
| **50 µg** |  |  |  |  |
| Specific gravity | 1.015 | 1.015 | 1.015 | 1.020 |
| pH | 7.0 | 6.5 | 7.5 | 6.5 |
| Appearance | clean/transparent | clean/transparent | clean/transparent | clean/transparent |
| Protein | - | - | - | - |
| Glucose | - | - | - | - |
| Bilirubin | - | - | - | - |
| Ketones | - | - | - | - |
| Leukocytes | - | - | - | - |
| Urobilinogen | - | - | +/- | - |

-, negative, +/-; slightly positive; + to ++++, increasing degrees of positivity.

Supplementary Table S4: Urine analysis of male minipigs treated with StreptInCor at the concentration of 100 µg/500 µL or 200 µg/500 µL.

| Animal (male) | #1 | #2 | #3 | #4 |
| --- | --- | --- | --- | --- |
| **100 µg** |  |  |  |  |
| Specific gravity | 1.015 | 1.015 | 1.020 | 1.020 |
| pH | 8.5 | 6.5 | 7.0 | 7.0 |
| Appearance | clean/transparent | clean/transparent | clean/transparent | clean/transparent |
| Protein | - | - | - | - |
| Glucose | - | - | - | - |
| Bilirubin | - | - | - | - |
| Ketones | - | - | - | - |
| Leukocytes | - | - | - | - |
| Urobilinogen | +/- | - | - | - |
|  |  |  |  |  |
| **200 µg** |  |  |  |  |
| Specific gravity | 1.015 | 1.025 | 1.015 | 1.020 |
| pH | 8.0 | 7.0 | 6.5 | 6.5 |
| Appearance | clean/transparent | clean/transparent | clean/transparent | clean/transparent |
| Protein | - | - | - | - |
| Glucose | + | - | - | - |
| Bilirubin | - | - | - | - |
| Ketones | - | - | - | - |
| Leukocytes | - | - | - | - |
| Urobilinogen | - | - | - | +/- |

-, negative, +/-; slightly positive; + to ++++, increasing degrees of positivity.

Supplementary Table S5. Urine analysis of female minipigs treated with placebo or StreptInCor at the concentration of 50 µg/500 µL

| Animal (female) | #1 | #2 | #3 | #4 |
| --- | --- | --- | --- | --- |
| **Placebo** |  |  |  |  |
| Specific gravity | 1.020 | 1.025 | 1.015 | 1.020 |
| pH | 6.5 | 6.0 | 8.0 | 7.5 |
| Appearance | clean/transparent | slightly cloudy | clean/transparent | clean/transparent |
| Protein | - | - | - | - |
| Glucose | - | - | - | - |
| Bilirubin | - | - | - | - |
| Ketones | - | - | - | - |
| Leukocytes | - | - | - | - |
| Urobilinogen | - | +/- | - | - |
|  |  |  |  |  |
| **50 µg** |  |  |  |  |
| Specific gravity | 1.015 | 1.015 | 1.020 | 1.025 |
| pH | 6.5 | 7.5 | 8.0 | 8.0 |
| Appearance | clean/transparent | clean/transparent | clean/transparent | clean/transparent |
| Protein | - | - | - | - |
| Glucose | - | - | - | - |
| Bilirubin | - | - | - | - |
| Ketones | - | - | - | - |
| Leukocytes | - | - | - | - |
| Urobilinogen | - | +/- | - | - |

-, negative, +/-; slightly positive; + to ++++, increasing degrees of positivity.

Supplementary Table S6: Urine analysis of male minipigs treated with StreptInCor at the concentration of 100 µg/500 µL or 200 µg/500 µL.

| Animal (female) | #1 | #2 | #3 | #4 |
| --- | --- | --- | --- | --- |
| **100 µg** |  |  |  |  |
| Specific gravity | 1.020 | 1.025 | 1.015 | 1.020 |
| pH | 6.5 | 6.0 | 7.0 | 8.0 |
| Appearance | clean/transparent | clean/transparent | clean/transparent | clean/transparent |
| Protein | - | - | - | - |
| Glucose | - | - | - | - |
| Bilirubin | - | + | - | - |
| Ketones | - | - | - | - |
| Leukocytes | - | - | - | - |
| Urobilinogen | - | +/- | - | - |
|  |  |  |  |  |
| **200 µg** |  |  |  |  |
| Specific gravity | 1.020 | 1.025 | 1.025 | 1.015 |
| pH | 7.0 | 6.0 | 7.5 | 7.0 |
| Appearance | clean/transparent | clean/transparent | clean/transparent | clean/transparent |
| Protein | - | - | - | - |
| Glucose | - | - | + | - |
| Bilirubin | - | - | - | - |
| Ketones | - | - | - | - |
| Leukocytes | - | - | - | - |
| Urobilinogen | - | - | - | - |

-, negative, +/-; slightly positive; + to ++++, increasing degrees of positivity.

Supplementary Figure S2. Evaluation of humoral immune responses against StreptIncor





Supplementary Figure S2. Evaluation of humoral immune responses against StreptIncor of male (a) and female (b) minipigs treated with placebo or increasing concentrations of StreptInCor in the formulations of the GAS adsorbed vaccine (50, 100, or 200 µg/500 µL).

Supplementary Figure S3. Mitral valve of the male minipig treated with StreptInCor


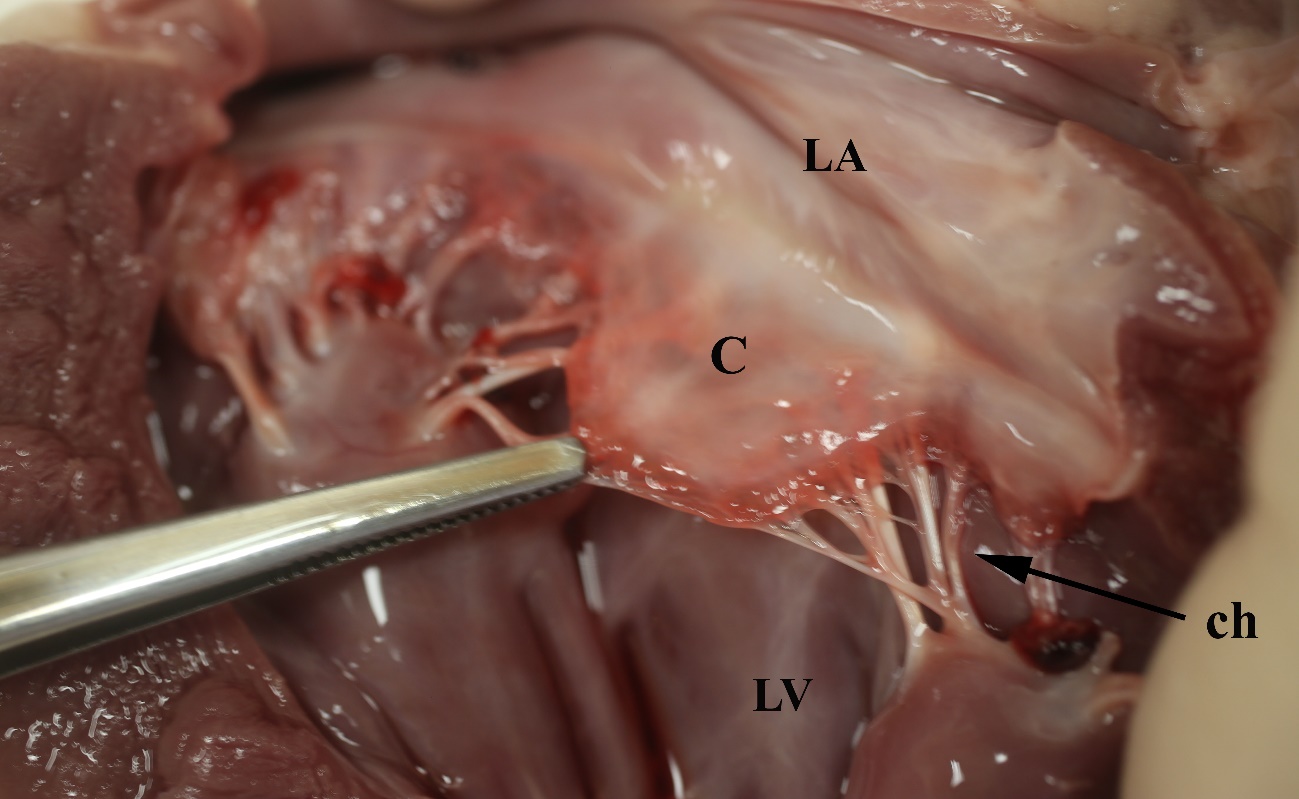


Supplementary Figure S3. Mitral valve of the male minipig treated with StreptInCor (100 µg/500 µL) showing no gross alterations. C= anterior cusp; ch= tendinous cords; LA= left atrium and LV= left ventricle.

Supplementary Figure S4. Mitral valve of a minipig control.


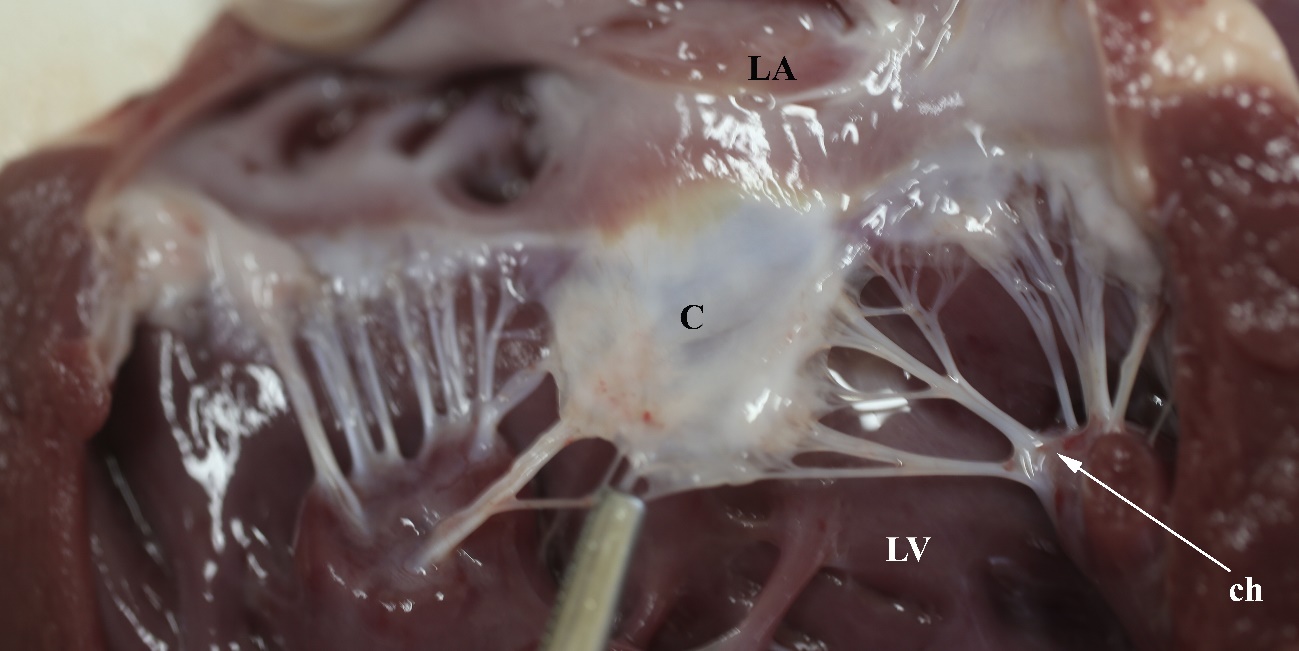


Supplementary Figure S4. Mitral valve of a minipig control treated with placebo showing no gross alterations. C= anterior cusp; ch= tendinous cords; LA= left atrium and LV= left ventricle.

Supplementary Figure S5: Photomicrographs of histological sections of kidney and liver from minipigs treated with placebo.


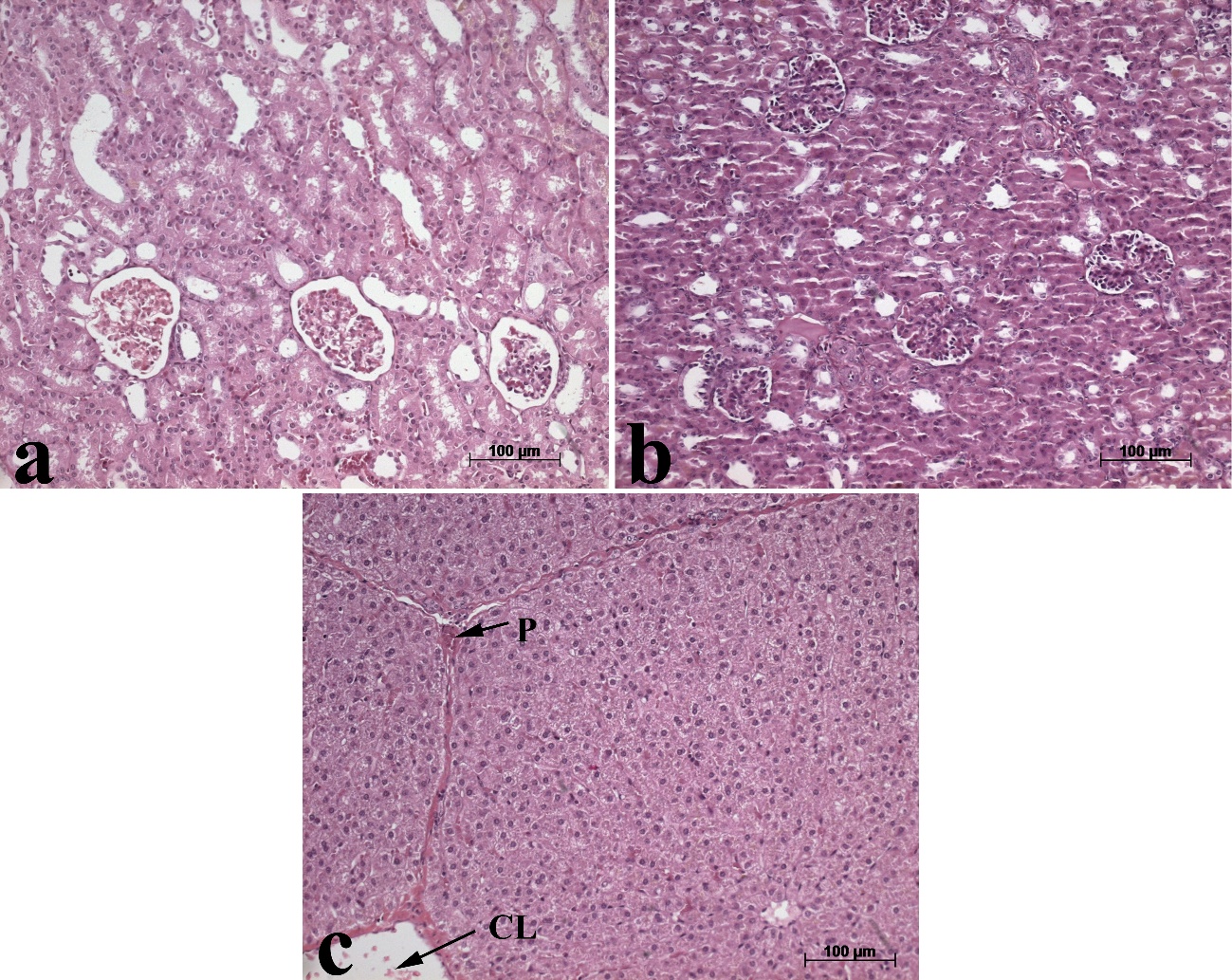


Supplementary Figure S5. Photomicrographs of histological sections of left (a) and right (b) kidneys from a female minipig and liver (c) from a male animal, both treated with placebo (control group). In (c), there was a minimal congestion in portal (P) and centrilobular veins (CL) (arrows). No other histopathological alterations were observed in renal or hepatic tissues.

Supplementary Video S1. Echocardiography from minipigs before injection of GAS adsorbed vaccine.

Supplementary Video S2. Echocardiography from minipigs after injection of GAS adsorbed vaccine.
